# The influence of outdoor physical activity on the physical and mental health of college students: a systematic review

**DOI:** 10.3389/fpsyg.2026.1898808

**Published:** 2026-09-15

**Authors:** Jianfeng Fu, Yanchao Zhang, Zeng Cheng

**Affiliations:** College of Basic Education, Guangxi University of Finance and Economics, Nanning, Guangxi, China

**Keywords:** college students, outdoor physical activity, physical and mental health, review, systematic

## Abstract

**Objective:**

Outdoor activity plays a significant role in promoting physical and mental health, but the impact of outdoor physical activity on college students’ physical and mental health remains unclear. Clarifying this relationship will inform mental health interventions and the design of campus activities. Therefore, this study aims to systematically investigate the influence of outdoor physical activity on college students’ physical and mental health.

**Methods:**

A systematic search was conducted in the PubMed, EBSCO, and Web of Science databases for English-language literature published from the inception of each database through March 2026. The literature screening process consisted of three stages: removal of duplicates, title and abstract screening, and full-text review. The entire study strictly followed the PRISMA guidelines, and the methodological quality of the included studies was assessed using the Cochrane Risk of Bias Assessment Tool.

**Results:**

This study ultimately included 32 studies involving a total of 7,274 university students. It was established that outdoor physical activity has positive influences on multiple psychological dimensions among university students, including depression, stress, emotional state, vitality, self-esteem, well-being, and academic burnout. However, no consistent conclusions have been reached regarding the effects of outdoor physical activity on anxiety and attention. Additionally, outdoor physical activity has a positive influence on enhancing physical activity levels and promoting physical health among university students.

**Conclusion:**

Outdoor physical activity can influence the physical and mental health of college students. However, the current number of studies examining the influence of outdoor physical activity on physical activity levels and physical health among college students is relatively limited, and related conclusions should be interpreted with caution. Future research should adopt more rigorous study designs and further examine the effects of different forms of outdoor physical activity on the physical health of college students.

## 1 Introduction

College students are at a critical developmental stage as they transition from late adolescence to early adulthood, a period during which lifestyle habits and health-related behaviors are formed. However, the combined effects of academic competition, employment pressure, financial strain, and challenges in adapting to interpersonal relationships and social roles have made mental health problems increasingly salient in college students. Previous studies indicate that symptoms of mental disorders are highly prevalent among college students, with depression and anxiety affecting approximately 35.41% and 40.21%, respectively, and suicidal ideation within the past 12 months reported by up to 10.76% (Paiva et al., 2025). These mental health issues severely impair college students’ academic performance and quality of life (Bruffaerts et al., 2018; Freitas et al., 2023). They also impose a heavy economic burden on society. Studies show that globally, the economic losses caused by common mental health issues such as depression and anxiety amount to approximately $1 trillion annually (Chisholm et al., 2016).

Extensive research indicates that higher levels of physical activity are generally associated with lower levels of depression, anxiety, and psychological distress, as well as higher levels of well-being; physical activity interventions have also been shown to improve the mental health of college students to some extent (Rodríguez-Romo et al., 2022; Li et al., 2024). Although college students have greater behavioral autonomy compared to high school students, the constraints of mandatory physical education classes and parental supervision are correspondingly reduced, leading to a decrease in external support and opportunities for physical activity. Moreover, heavy academic workloads, limited discretionary time, inadequate motivation to exercise, and restricted access to exercise facilities may further hinder college students’ participation in physical activity (Ferreira Silva et al., 2022). Physical inactivity has become a widespread health issue among college students and has a significant impact on their physical and mental well-being. Insufficient physical activity may impair an individual’s ability to regulate emotions, thereby increasing the risk of emotional disorders such as depression and anxiety (Li et al., 2023). Therefore, it is necessary to implement effective interventions to increase physical activity levels among college students and further promote their mental health.

Outdoor physical activity, as a health promotion approach that combines the benefits of exercise with exposure to the natural environment, has garnered increasing attention in recent years. Compared with general physical activity, outdoor physical activity not only enhances cardiopulmonary function, physical fitness, and emotional well-being through exercise itself but also confers additional physical and mental health benefits through exposure to nature and sunlight, enhanced recovery experiences, and greater enjoyment of exercise (Kljajević et al., 2021; Huang et al., 2024; Noseworthy et al., 2023; Wicks et al., 2022). Engaging in physical activity in natural environments may enhance participants’ enjoyment and willingness to remain active, thereby supporting sustained participation in physical activity (Thompson Coon et al., 2011; Peddie et al., 2024). For college students who spend prolonged periods in classrooms, libraries, dormitories, and in front of electronic screens, outdoor physical activity not only provides additional opportunities for exercise but also allows them to temporarily disengage from cognitively demanding academic environments, restore attentional resources, and alleviate academic stress (Bramwell et al., 2023; Shrestha et al., 2021). At the same time, a university campus is not a community environment in the conventional sense, but rather a “micro-living environment” that integrates learning, living, commuting, socializing, leisure, and physical activity, characterized by relative concentration and institutionalized management. Existing research indicates that campus walking environments, road conditions, and physical activity facilities are closely associated with university students’ physical activity levels, while campus green spaces, blue spaces, and natural landscapes may help promote psychological recovery, improve mental health, and enhance quality of life (Murphy et al., 2024; Ding et al., 2024). Moreover, university campuses offer diverse outdoor activity spaces, centrally located sports facilities, and well-established organizational support systems. These features make outdoor physical activity accessible, flexible in format, and easily integrated into students’ daily academic lives. Therefore, outdoor physical activity may represent a feasible strategy for promoting the physical and mental health of college students.

Although existing studies have reviewed the role of outdoor physical activity in promoting physical and mental health, there remains a lack of systematic evidence synthesis specifically targeting university students. First, a review published by Lahart et al. (2019) examined the effects of green exercise on physical and mental health, noting that compared to exercise in non-natural environments, green exercise may offer certain advantages in improving mood and enhancing exercise enjoyment. However, the overall evidence remains limited by a high risk of bias and significant study heterogeneity, and lacks specific findings for university students (Lahart et al., 2019). Second, a 2020 review by Wicks et al. primarily focused on the differences in the effects of outdoor physical activity in natural versus urban environments on mental health. The results indicated that outdoor physical activity in natural environments may offer greater benefits in alleviating anxiety and fatigue, as well as enhancing positive emotions and vitality. However, this study primarily focused on mental health outcomes without systematically addressing physical health outcomes, and the study population did not consist of university students (Wicks et al., 2022). Furthermore, although the 2023 review by Noseworthy et al. (2023) compared the effects of outdoor versus indoor exercise on mental health, physical health, and physical activity behaviors, the included participants were still a general population, making it insufficient to provide targeted evidence to directly support the design of relevant interventions in a university campus setting. It is worth noting that in recent years, research on the health effects of outdoor physical activity, green exercise, and physical activity in natural settings has continued to grow, and relevant evidence regarding university campuses and young people has also been accumulating. However, to date, there remains a lack of a systematic review specifically focused on university students that comprehensively integrates the effects of outdoor physical activity on both mental and physical health outcomes. Based on this, this study aims to employ a systematic review methodology to comprehensively examine the impact of outdoor physical activity on the physical and mental health of university students, to provide a reference for the development of health intervention measures and the design of campus physical activity programs.

## 2 Methods

This study was conducted and reported in strict accordance with the PRISMA reporting guidelines to enhance the transparency of the research process and the completeness of reporting (Moher et al., 2015). This systematic review was not registered on the Prospective Registration of Systematic Reviews (PROSPERO) platform. Before literature screening and data extraction were initiated, the research team reached consensus on the research question, inclusion and exclusion criteria, search strategy, screening procedures, data extraction items, and quality assessment methods. These procedures are described in detail in the Methods section of this paper.

### 2.1 Literature sources and search strategies

To systematically identify studies examining the effects of outdoor physical activity on the physical and mental health of college students, we searched three electronic databases, namely PubMed, EBSCO, and Web of Science, from their respective dates of inception through March 23, 2026. The search strategy was organized into the following three conceptual clusters based on the research questions: outdoor or natural environments, physical activity, and mental health. Search terms within the same conceptual cluster were linked using “OR,” while terms across different clusters were linked using “AND.” The main search terms included: Outdoor (outdoor* OR outside* OR park* OR greenspace* OR “green space*” OR bluespace* OR “blue space*” OR “natural environment*” OR nature OR forest*); Outdoor exercise (“green exercis*” OR “green gym” OR “blue exercis*” OR “blue gym” OR ecotherap*); Physical activity (exercis* OR “physical activit*” OR walk* OR “physical fit*” OR run OR runs OR running OR runner* OR athlet*); Mental Health (“mental health” OR mood OR “well-being” OR wellbeing OR “well being” OR “self-esteem” OR depress* OR anxiety OR stress OR affect OR emotion* OR “quality of life” OR psycholog*). The search queries described above were adapted to the subject heading systems, field specifications, truncation conventions, and search syntax of each database. The initial search strategy prioritized sensitivity by using relatively broad terms related to outdoor environments, physical activity, and health outcomes, thereby minimizing the risk of excluding potentially relevant studies. However, terms such as “outdoor*,” “outside*,” “park*,” “greenspace*,” “green space*,” “bluespace*,” “blue space*,” “natural environment*,” “nature” and “forest*” encompass a wide range of contexts. Consequently, the search retrieved a substantial number of studies that focused exclusively on exposure to natural environments or access to green spaces, did not examine outdoor physical activity, or did not include college students as the study population.

### 2.2 Literature screening process

*Inclusion criteria*: (1) The study population consists of college students; (2) The exposure factor or intervention examined in the study is physical activity conducted in an outdoor environment; (3) Study outcomes include physical and/or mental health indicators; (4) The study examines the impact of outdoor physical activity on physical and/or mental health as outcome variables; (5) Published in English in a journal and (6) Publication date up to March 23, 2026.

*Exclusion criteria*: (1) Study participants were not college students; (2) Studies that examined only exposure to natural environments, green space accessibility, or contact with nature, without including physical activity; (3) Physical activity took place indoors, or it was impossible to determine whether the activity occurred in an outdoor setting; (4) Studies that did not report physical activity or mental health outcomes; (5) Studies that only described college students’ attitudes, preferences, or willingness to participate in outdoor physical activity; (6) Conference abstracts, theses, research protocols, reviews, commentaries, editorials, book chapters, or other non-peer reviewed literature; (7) Full-text articles that were unavailable or lacked sufficient key information to determine eligibility and (8) Non English language literature.

All retrieved records were imported into EndNote for reference management. Duplicate records were removed through a combination of automated detection and manual verification. Two reviewers (JF and YZ) then independently screened the titles and abstracts of all remaining records against the predefined inclusion and exclusion criteria. At this stage, records were excluded if they involved ineligible populations, addressed topics unrelated to outdoor physical activity, did not assess physical activity or psychological outcomes, or represented ineligible publication types. When the information provided in the title and abstract was insufficient to determine eligibility, or when a record appeared potentially eligible, the full text was retrieved and independently assessed by both reviewers. During full-text screening, one primary reason for exclusion was documented for each excluded study. These reasons included an ineligible study population, the absence of an outdoor physical activity component, failure to report an eligible health outcome, failure to analyze the association between outdoor physical activity and health outcomes, and an ineligible publication type. Any disagreements between the two reviewers were resolved through discussion; when consensus could not be reached, a third reviewer (ZC) was consulted to make the final decision. The study selection process was documented using the PRISMA 2020 flow diagram, which reported the number of records identified from each database, the number remaining after duplicate removal, the number screened by title and abstract, the number assessed in full text, the number excluded at the full-text stage and the corresponding reasons for exclusion, and the final number of included studies. The high initial search yield was primarily attributable to the sensitivity-oriented search strategy, as several broad search terms retrieved numerous studies focusing solely on natural-environment exposure or populations other than college students. Conversely, the relatively small number of studies ultimately included reflected the simultaneous application of eligibility criteria relating to college students, outdoor environments, physical activity, and psychological outcomes, rather than any *post hoc* narrowing of the review scope during study selection.

### 2.3 Data extraction

Two researchers (JF and YZ) independently extracted data from the included studies and entered the relevant information into a predesigned standardized data extraction form. To ensure the accuracy and consistency of data extraction, a third researcher (ZC) conducted random checks and reviewed the extracted data. Any disagreements were resolved through discussion and consensus. The extracted data mainly included authors, year of publication, country of origin, study design, sample size, intervention characteristics, and study findings.

### 2.4 Assessment of risk of Bias

To evaluate the methodological quality and potential risk of bias in the included studies, two researchers (JF and YZ) independently conducted a risk of bias assessment. For randomized controlled trials, the Cochrane Risk of Bias Assessment Tool was used (Jørgensen et al., 2016). This tool consists of seven questions to assess the risk of bias and classifies studies as “low risk,” “unclear” or “high risk.” The seven questions are: (1) random sequence allocation; (2) allocation concealment; (3) blinding of participants and personnel; (4) blinding of outcome assessment; (5) missing outcome data; (6) selective reporting; and (7) other sources of bias. The two reviewers assessed all articles included in the study. Disagreements regarding the assessment were resolved through discussion with a third reviewer (ZC).

## 3 Results

A total of 37,210 relevant records were retrieved from three databases: PubMed, EBSCO, and Web of Science. After removing duplicates, 27,947 articles remained for title and abstract screening. Subsequently, we conducted full-text reviews and eligibility assessments of the articles that appeared to meet the inclusion criteria following the initial screening. Ultimately, 32 studies were included in the systematic review. Figure 1 illustrates the literature screening process for this study.

**Figure 1:**
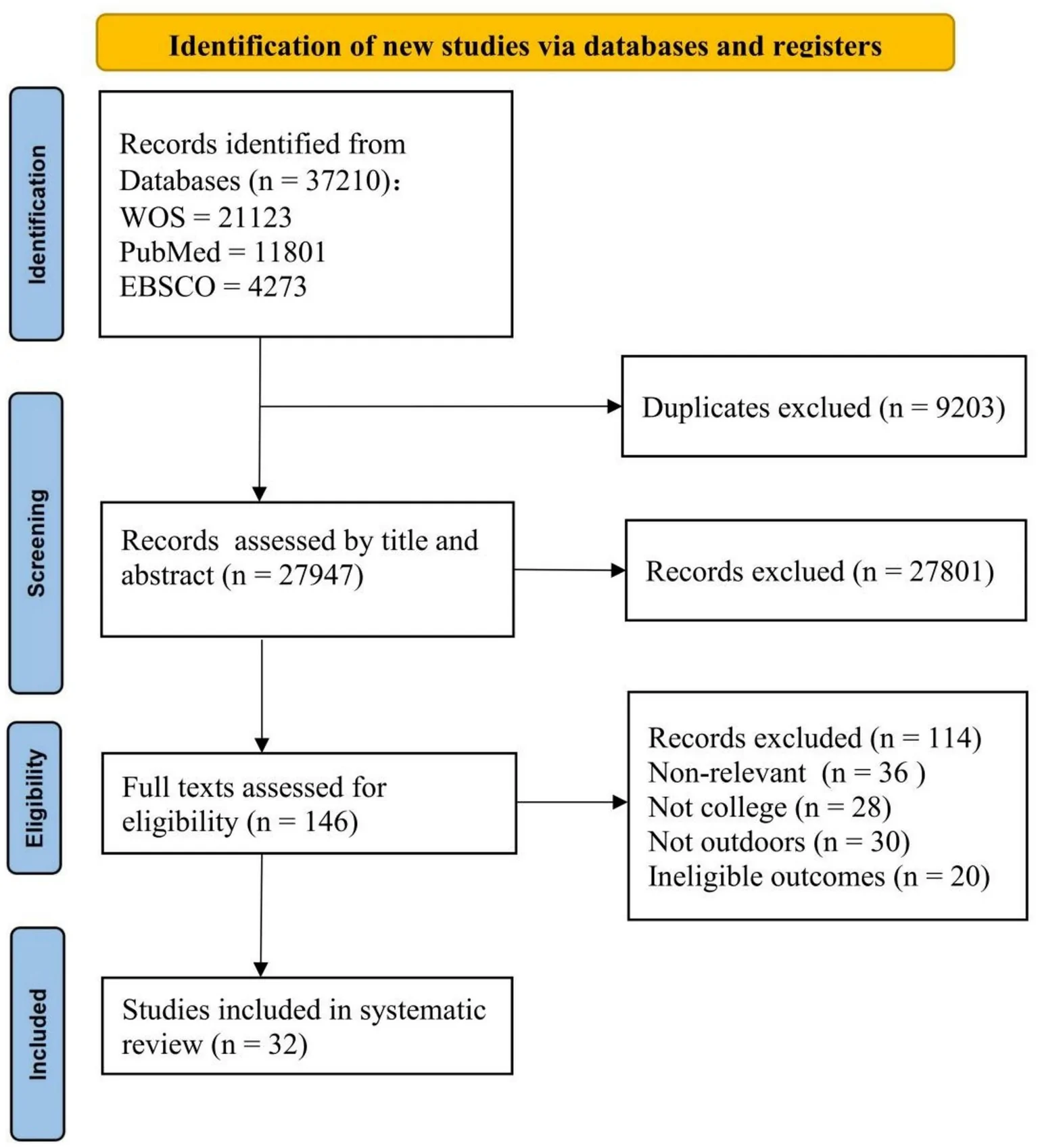
Literature inclusion and exclusion process.

### 3.1 Study characteristics

#### 3.1.1 Participant characteristics

Detailed information on the included studies is presented in Table 1. This systematic review ultimately included 32 studies involving a total of 7,274 participants. The average age of participants ranged from 17 to 28 years. In terms of geographical distribution, the included studies spanned multiple countries and regions, with the largest number of studies conducted in China (*n* = 9) (Han and Wang, 2018; Han, 2017; Jiang et al., 2025; Liu X. et al., 2025; Liu T. et al., 2025; Mao et al., 2022; Wang et al., 2021; Xu et al., 2026; Xu, 2025), followed by the United States (*n* = 7) (Bramwell et al., 2023; Fuegen and Breitenbecher, 2018; Focht, 2009; Olson et al., 2024; Plante et al., 2007; Plante et al., 2003; Plante et al., 2006) and Japan (*n* = 4) (Kerr et al., 2006; Song et al., 2014; Song et al., 2015; Song et al., 2013); the remaining studies were from the United Kingdom (*n* = 3) (Flowers et al., 2018; Ma et al., 2023; Wyles et al., 2017), South Korea (*n* = 2) (Bang et al., 2017; Kim et al., 2021), Switzerland (*n* = 1) (Johansson et al., 2011), France (*n* = 1) (Legrand et al., 2022), Ireland (*n* = 1) (Shrestha et al., 2021), Italy (*n* = 1) (Poli et al., 2024), Luxembourg (*n* = 1) (Olafsdottir et al., 2020), Iceland (*n* = 1) (Olafsdottir et al., 2017), and Spain (*n* = 1) (Pérez-Conde et al., 2025).

**Table 1:** Characteristics of the included studies.

Study	Country	Age	Study design	Sample	Duration	Interventions	Results
Bang et al. (2017)	South Korea	24.3 ± 4.19	Quasi-experimental	118	Six week	Forest Walk	Compared with the control group, the intervention significantly increased participants’ physical activity levels and significantly reduced depression.
Bramwell et al. (2023)	America	18–24	Randomized controlled	13	Four week	Outdoor group sports courses and indoor sports courses	The outdoor exercise group experienced a more significant reduction in perceived stress than the indoor exercise group
Flowers et al. (2018)	England	19.9 ± 4.26	Randomized controlled	60	Five month	Outdoor cycling, indoor cycling, and watching green sports videos	Research findings indicate that outdoor exercise provides greater psychological benefits than indoor exercise, and these benefits can be enhanced through expectation-adjustment.
Fuegen and Breitenbecher (2018)	America	21.59 ± 7.69	Randomized controlled	181	/	Indoor exercise (walking), indoor rest, outdoor exercise (walking), outdoor rest	Outdoor activity can improve mood, boost energy levels, and strengthen willpower. Participants reported lower levels of fatigue compared to those who rested or stayed indoors. However, in terms of changes in attention or negative effects, they did not differ significantly from participants who rested or attended meetings indoors.
Focht (2009)	America	22.14 ± 1.73	Crossed Experimental	35	/	Outdoor walks and lab walks	A brief walk in either setting led to improvements in emotional well-being. Our findings show that the outdoor walking group experienced significant increases in general well-being, perceived arousal, revitalization, and engagement. Participants also reported higher ratings of enjoyment and a greater willingness to participate in the future following the outdoor walk.
Han and Wang (2018)	China	20.47 ± 1.23	Randomized controlled	95	Three month	Green Sports and Social Interaction	The level of physical activity associated with high-intensity green exercise is significantly higher than that associated with low-intensity green exercise.
Han (2017)	China	20.84 ± 1.14	Randomized controlled	116	Five month	Walking and running in natural settings; walking and running in urban environments.	College students who engage in activities in natural environments experience lower levels of fatigue and anxiety and can improve their concentration.
Johansson et al. (2011)	Switzerland	22.4 ± 3.03	Randomized controlled	20	Five week	Walking in the city, walking in the park	The sense of time pressure decreases when walking in a park rather than when walking on the street. Walking alone in a park leads to a greater sense of rejuvenation, but walking with friends on the street leads to an even greater sense of rejuvenation.
Jiang et al. (2025)	China	20.26 ± 1.22	Cross-sectional study	418	/	/	Engaging in outdoor physical activity in green spaces is associated with better mental health, while engaging in physical activity in green spaces at night is linked to lower anxiety levels and greater well-being.
Kerr et al. (2006)	Japan	21.67 ± 1.51	Crossed Experimental	22	/	Indoor running and outdoor running	Concerns about the impact of running environments on changes in emotional states may be overstated, as in the two current experiments, only one emotional state (a sense of pride) showed a significant difference between laboratory and natural running environments.
Kim et al. (2021)	South Korea	22.1 ± 1.6	Randomized controlled	38	Three month	Forest Activities	Participants in the campus forest intervention exhibited significantly improved positive emotional states, reduced stress responses, and increased well-being following the intervention. The findings of this study indicate that campus forest interventions are effective in improving college students’ mental health.
Legrand et al. (2022)	France	20.2 ± 1.3	Randomized controlled	150	/	Walking outdoors, walking indoors, and not exercising	Green exercise significantly increased positive effects and significantly reduced negative effects, whereas in urbanized metropolitan areas, the same exercise regimen only reduced negative effects
Liu X. et al. (2025)	China	18–28	Qualitative Study	11	/	/	Outdoor water sports can improve college students’ mental health.
Liu T. et al. (2025)	China	20.3 ± 1.4	Cross-sectional study	1,022	/	/	Green exercise is directly associated with lower levels of emotional exhaustion, cynicism, and reduced personal accomplishment, while nature-based connectedness partially mediates these effects, accounting for a significant proportion of the overall association.
Ma et al. (2023)	England	23.6	Randomized controlled	104	Four month	Outdoor walking	Outdoor walks can improve college students’ sleep quality, mood, and levels of mindfulness.
Mao et al. (2022)	China	18–19	Before-and-after study	2,852	/	Outdoor jogging	Jogging outdoors in campus green spaces is closely linked to physical health.
Olafsdottir et al. (2020)	Luxembourg	24.39 ± 2.61	Randomized controlled	90	Three month	Walking in nature, walking at the gym, observing the natural environment in the lab	Taking a walk in nature is more effective at reducing stress responses than exercising alone or simply viewing natural scenes.
Olson et al. (2024)	America	/	Before-and-after study	28	/	Outdoor walking	Participants’ well-being increased significantly, while their resilience, anxiety, and perceived stress remained largely unchanged during periods of stress and challenge.
Olafsdottir et al. (2017)	Iceland	/	Randomized controlled	90	Four month	Walking in nature, walking at the gym, observing the natural environment in the lab	Exercising outdoors can help reduce stress levels.
Plante et al. (2007)	America	17–23	Randomized controlled	128	/	Sports in different environments (i.e., laboratories, indoor fitness clubs, outdoors)	Outdoor activities can help improve women’s mental health.
Plante et al. (2003)	America	/	Randomized controlled	154	/	Outdoor brisk walking; a combination of virtual reality and walking on a treadmill in the laboratory; virtual reality alone; control group	Women generally derive greater emotional benefits from outdoor exercise. Compared to the male sample, the environment may have positively enhanced the psychological and emotional benefits of exercise among the female sample. The only positive benefit derived from the virtual reality environment was an increase in the women’s sense of calm.
Pérez-Conde et al. (2025)	Spanish	21.76 ± 4.85	Randomized controlled	106	Twelve week	A 1-h hike in the mountains and 1 h of strength and resistance training outdoors	Outdoor physical activities significantly reduced academic burnout—particularly emotional exhaustion and stress levels—and increased daily step counts.
Poli et al. (2024)	Italy	24.3 ± 3.2	Randomized controlled	31	Two month	Enhanced Reality Cycling, Virtual Reality Cycling, Outdoor Cycling	VRC and ERC enhance engagement and physiological responses during indoor cycling, but outdoor cycling offers greater benefits in terms of motivation, enjoyment, and future participation intentions.
Plante et al. (2006)	America	/	Randomized controlled	112	/	Walking outdoors, walking on a treadmill in the lab while watching a virtual video of a campus walk, or watching a virtual walking video (without actually walking)	Outdoor activities can reduce fatigue and boost women’s energy levels.
Song et al. (2014)	Japan	21.2 ± 1.7	Crossed Experimental	17	/	Walking in the city, walking in the park	Heart rates were significantly lower during walks in urban parks than during walks on city streets. In addition, parasympathetic activity increased, and sympathetic activity decreased during walks in urban parks. After walking in urban parks, scores for the parameters “comfort,” “nature,” and “relaxation” were significantly higher. After walking in an urban park, energy levels increased significantly, while scores for negative emotions such as “tension and anxiety” and “fatigue” decreased significantly. After walking in an urban park, psychological reaction scores also decreased significantly.
Shrestha et al. (2021)	Ireland	24.92 ± 3.55	Qualitative Study	13	/	/	Compared to walking on urban streets, walking in natural settings has a more invigorating and refreshing effect, which is closely linked to positive sensory, emotional, and cognitive states.
Song et al. (2015)	Japan	22.3 ± 1.2	Randomized controlled	23	/	Walking in parks and walking in urban areas	A brief walk in a city park can stimulate parasympathetic activity, inhibit sympathetic activity, lower heart rate, improve mood, and reduce anxiety.
Song et al. (2013)	Japan	22.5 ± 3.1	Randomized controlled	13	/	Walking in the City Park	Participants reported feeling more comfortable, at ease, relaxed, and energetic while walking in urban parks than while walking in downtown areas. In addition, their levels of negative emotions and anxiety were significantly lower.
Wyles et al. (2017)	England	22 ± 6.18	Randomized controlled	90	/	Beach cleanup, rock pools, and coastal walks	All three coastal activities are associated with positive emotions and pro-environmental intentions.
Wang et al. (2021)	China	18–21	Randomized controlled	77	Four month	Brisk walking in the park, leisurely walking in the park, brisk walking at the gym, and leisurely walking at the gym	When it comes to reducing stress and improving concentration among obese individuals, the park environment is often more effective than indoor gyms.
Xu et al. (2026)	China	21.9	Quasi-experimental	71	/	Indoor training and outdoor training in green spaces	Regular outdoor training in green spaces can significantly reduce anxiety related to athletic competition
Xu (2025)	China	/	Cross-sectional study	976	/	/	This study found that, among college students, outdoor exercise is associated with lower levels of academic burnout, and social support and self-regulation account for a statistically significant proportion of this association, including the sequential pathway linking these two mediators.

#### 3.1.2 Study design characteristics

The 32 included studies employed a variety of study designs, with randomized controlled trials being the most common (*n* = 20). Other designs included randomized crossover trials (*n* = 3), cross-sectional studies (*n* = 3), quasi-experimental studies (*n* = 2), qualitative studies (*n* = 2), and before-and-after studies (*n* = 2). Regarding intervention types, outdoor walking was the most common intervention, while other interventions included outdoor running, exercise in green spaces, outdoor cycling, outdoor group exercise, and forest activities.

### 3.2 Risk of Bias assessment

The results of the risk of bias assessments for the included studies are shown in Figures 2, 3.

**Figure 2:**
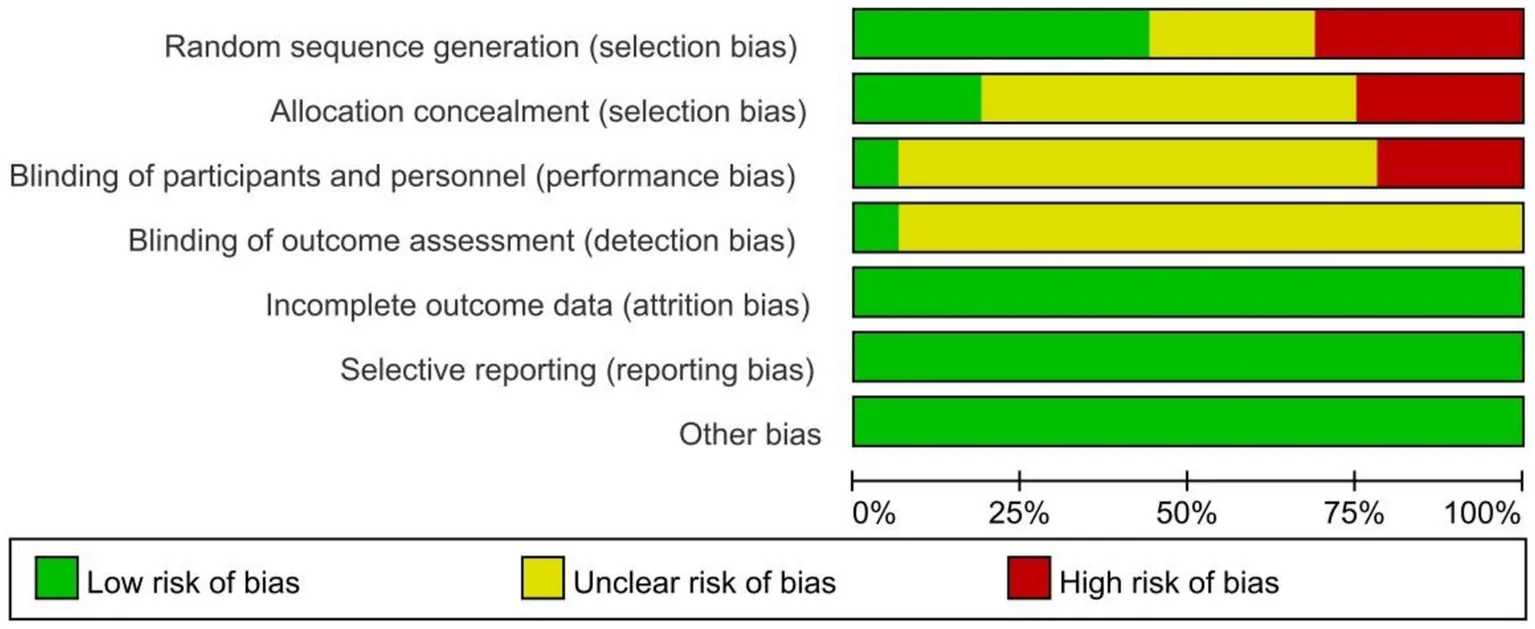
Risk of bias summary across each risk of bias item.

**Figure 3:**
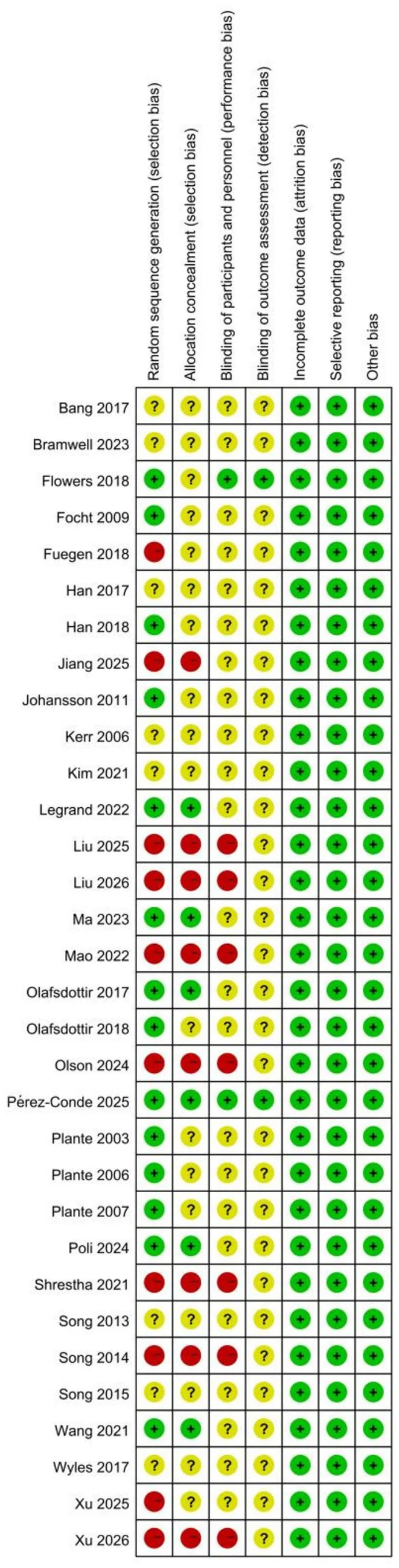
Risk of bias summary for each included study: + denotes low risk of bias; − denotes high risk of bias;?denotes unclear risk of bias.

### 3.3 Results of the systematic review

#### 3.3.1 Effects of outdoor physical activity on mental health

##### 3.3.1.1 Depression

Three studies found that four types of outdoor physical activity had an impact on depression symptoms among college students (Jiang et al., 2025; Bang et al., 2017; Pérez-Conde et al., 2025). Specifically, forest walks, outdoor physical activity, outdoor walks, and outdoor strength training were all found to significantly improve depression symptoms in college students.

##### 3.3.1.2 Anxiety

Regarding anxiety, existing research findings are not entirely consistent. Two studies indicated that outdoor physical activity can improve anxiety symptoms among college students, including outdoor water sports and general outdoor physical activity (Jiang et al., 2025; Liu X. et al., 2025). In addition, training in outdoor green spaces can also reduce competition anxiety among college athletes (Xu et al., 2026). However, three other studies found that outdoor walking and outdoor strength training did not improve anxiety in college students (Olson et al., 2024; Olafsdottir et al., 2017; Pérez-Conde et al., 2025).

##### 3.3.1.3 Stress

A total of six studies examined the effects of outdoor physical activity on stress. Five studies reported that outdoor team sports, outdoor walking, park walking, and outdoor strength training reduced perceived stress among college students (Wang et al., 2021; Xu, 2025; Olson et al., 2024; Olafsdottir et al., 2020; Olafsdottir et al., 2017; Pérez-Conde et al., 2025). However, a study by Kerr et al. (2006) found that outdoor running does not reduce stress among college students.

##### 3.3.1.4 Mood

Mood was one of the most frequently reported psychological outcomes in the included studies. Multiple studies indicate that outdoor running, forest activities, general outdoor exercise, outdoor walking, and brisk walking in parks can all improve the mood of college students (Han, 2017; Wang et al., 2021; Focht, 2009; Plante et al., 2007; Plante et al., 2003; Kerr et al., 2006; Ma et al., 2023; Kim et al., 2021). In addition, outdoor walking was found to reduce negative emotions (Johansson et al., 2011; Legrand et al., 2022).

##### 3.3.1.5 Vitality

Four studies reported on the effects of outdoor physical activity on vitality. The findings showed that outdoor walking increased vitality levels among female college students, while outdoor cycling and walking in parks also helped enhance vitality among college students (Plante et al., 2006; Song et al., 2014; Song et al., 2013; Flowers et al., 2018).

##### 3.3.1.6 Self-esteem

Only one study reported self-esteem outcomes, and its findings indicated that outdoor cycling enhanced self-esteem among college students (Flowers et al., 2018). Given the limited number of relevant studies, this finding requires further validation through additional research.

##### 3.3.1.7 Attention

Research findings regarding attention were mixed. Two studies indicated that outdoor walking did not significantly improve attention levels among college students (Fuegen and Breitenbecher, 2018). However, other studies found that outdoor walking, outdoor running, and walking in parks can improve attention performance among college students (Han, 2017; Mao et al., 2022; Johansson et al., 2011). Therefore, the effects of outdoor physical activity on attention remain inconsistent.

##### 3.3.1.8 Well-being

Four studies indicate that outdoor physical activity was associated with higher levels of well-being. Specifically, outdoor physical activity, outdoor walking, coastal walking, and forest activities all contribute to enhancing university students’ well-being levels (Murphy et al., 2024; Olson et al., 2024; Wyles et al., 2017; Kim et al., 2021). Overall, outdoor physical activity demonstrates a relatively consistent positive effect in promoting positive psychological experiences and subjective well-being.

##### 3.3.1.9 Academic burnout

Three studies reported on the effects of outdoor physical activity on academic burnout, with all results indicating that outdoor exercise can reduce academic burnout levels among college students (Liu T. et al., 2025; Xu, 2025; Pérez-Conde et al., 2025).

#### 3.3.2 Effects of outdoor physical activity on physical health

##### 3.3.2.1 Physical activity

Two studies examined the effects of outdoor physical activity on physical activity levels among university students. The findings showed that outdoor exercise increased physical activity levels among university students (Han and Wang, 2018; Bang et al., 2017).

##### 3.3.2.2 Physical health

Only one study reported physical health-related outcomes, and its findings indicated that outdoor jogging promoted physical health among university students (Mao et al., 2022). Due to the small number of studies involving this outcome, the relevant evidence remains limited.

#### 3.3.3 Effects of types of outdoor physical activity on college students’ physical and mental health

With respect to activity type, outdoor walking was the most frequently examined activity and was associated with the broadest range of psychological outcomes. Forest walks, general outdoor walks, and walks in parks have all been found to improve depressive symptoms, mood, vitality, well-being, and perceived stress (Han, 2017; Jiang et al., 2025; Wang et al., 2021; Xu, 2025; Song et al., 2014; Song et al., 2013; Wyles et al., 2017; Kim et al., 2021; Johansson et al., 2011; Legrand et al., 2022; Olafsdottir et al., 2020; Olafsdottir et al., 2017). However, the effects of outdoor walking on attention are inconsistent: some studies found that it improves attention performance (Han, 2017; Wang et al., 2021; Johansson et al., 2011), while others observed no significant changes (Fuegen and Breitenbecher, 2018); similarly, some studies reported that outdoor walking alleviates anxiety, whereas others found no significant effects (Olson et al., 2024; Olafsdottir et al., 2017). Collectively, these findings suggest that although walking-based activities may provide broad mental health benefits, their effects may vary according to the specific activity format, environmental characteristics, intervention intensity, and outcome measures used. In contrast, the evidence for activities such as running, strength training, cycling, and water sports is relatively limited, and findings across different psychological outcomes are not entirely consistent. Outdoor running improves mood and attention performance (Han, 2017; Focht, 2009; Plante et al., 2006; Kerr et al., 2006), but does not significantly reduce stress (Kerr et al., 2006); outdoor strength training improves depressive symptoms and reduces perceived stress, but has no significant effect on anxiety (Pérez-Conde et al., 2025). Outdoor cycling primarily enhances vitality and self-esteem (Flowers et al., 2018), while outdoor water sports and general outdoor physical activity can alleviate anxiety symptoms (Jiang et al., 2025; Liu X. et al., 2025). Furthermore, outdoor group sports have a positive impact on perceived stress (Xu, 2025), suggesting that activity formats characterized by social interaction may hold some value for stress management among college students.

#### 3.3.4 Effects of duration, frequency, and intensity of outdoor physical activity on college students’ physical and mental health

The studies included in this review varied substantially in activity duration, intervention frequency, and exercise intensity; however, few directly compared the effects of these parameters. Consequently, the optimal duration, frequency, and intensity of outdoor physical activity for improving college students’ mental health cannot yet be determined. Overall, walking-based activities-which are accessible and readily integrated into daily campus life-demonstrated beneficial effects across a broad range of psychological outcomes. By contrast, the effects of more physically demanding activities, such as running and strength training, appeared to be more outcome-specific.

#### 3.3.5 The impact of environmental characteristics of outdoor physical activity on college students’ physical and mental health

From an environmental perspective, physical activity undertaken in settings with prominent natural features, such as forests, parks, green spaces, and coastal areas, tended to produce more consistent benefits for psychological restoration. Forest activities can improve depressive symptoms, mood, and well-being (Kim et al., 2021); walking or brisk walking in parks helps enhance mood, vitality, and attention, and reduces subjective stress (Wang et al., 2021; Song et al., 2014; Legrand et al., 2022); and coastal walks are associated with increased well-being (Wyles et al., 2017). Furthermore, training in outdoor green spaces can reduce competition anxiety among college athletes (Xu et al., 2026). In contrast, while running, walking, and strength training in general outdoor settings have also been reported to produce some positive effects, their impact on stress, anxiety, and attention is more inconsistent.

## 4 Discussion

This study aimed to examine the effects of outdoor physical activity on the physical and mental health of college students. The results indicated that outdoor physical activity had positive effects on depression, stress, emotional state, vitality, self-esteem, well-being, and academic burnout among college students; however, findings regarding its effects on anxiety and attention were inconsistent. Notably, outdoor exercise increased physical activity levels among college students, whereas forest walking had no significant effect on physical activity. Furthermore, outdoor jogging was found to promote physical health in college students.

Outdoor walking, outdoor physical activity, and outdoor strength training were all found to significantly alleviate depressive symptoms and reduce stress levels among college students. Previous research has shown that walking in forests or green spaces can enhance feelings of relaxation and well-being, promote psychological restoration, and thereby help alleviate depressive mood and psychological stress (Bang et al., 2017; Chang et al., 2024; Yeon et al., 2021). At the same time, the mental health benefits of outdoor physical activity may arise from the combined effects of exposure to natural environments and exercise-induced physiological stimulation. On the one hand, green environments can reduce negative emotions such as anxiety, tension, anger, depression, and fatigue. On the other hand, physical activity can modulate neurotransmitters such as endorphins, endocannabinoids, dopamine, and serotonin, thereby improving emotional processing and reward-related experiences and ultimately alleviating depressive symptoms and psychological stress (Li et al., 2022; Heijnen et al., 2016). Furthermore, resistance training has been shown to effectively alleviate depressive symptoms (Gordon et al., 2018; O'Sullivan et al., 2023). Therefore, in addition to the general mental health benefits of physical activity, outdoor strength training may further improve college students’ psychological well-being by enhancing physical fitness, self-efficacy, and a sense of bodily control. However, the finding that outdoor jogging did not reduce stress levels among college students may be related to factors such as exercise intensity, duration of the intervention, individual adaptation, and characteristics of the outdoor environment. Future research should systematically compare the effects of different types, intensities, and durations of outdoor physical activity on mental health outcomes. At the same time, a multi-group randomized controlled design could be employed, with separate groups for indoor exercise, outdoor exercise in general built environments, and outdoor exercise in natural environments, to distinguish the independent effects of exercise stimuli, outdoor exposure, and natural environmental characteristics.

Outdoor walking, running, and cycling were all found to improve emotional well-being and enhance vitality among college students. Compared with indoor environments, natural settings typically provide green spaces, open views, natural light, and a reduced sense of environmental confinement, which may help alleviate attentional fatigue, reduce negative rumination, and promote emotional restoration and positive mood enhancement (Ohly et al., 2016; Li et al., 2022). Furthermore, outdoor activity provides richer sensory stimulation and a more expansive spatial experience, making it easier for individuals to feel relaxed, free, and in control; these positive subjective experiences may further enhance individual vitality (Shrestha et al., 2021; Puhakka, 2021). At the same time, research by Ryan et al. also indicates that outdoor activity and exposure to natural environments are closely associated with higher levels of subjective vitality (Ryan et al., 2010). Overall, outdoor physical activity may improve college students’ emotional states and enhance their vitality levels through the restorative effects of natural environments and the positive experiences gained during exercise.

Outdoor physical activity may enhance well-being and self-esteem among college students. It may help students temporarily disengage from prolonged academic stress and monotonous study environments, thereby improving their overall emotional state through the pleasurable and relaxing effects of exercise (Puhakka, 2021). In this process, individuals not only receive immediate positive emotional feedback but may also enhance their satisfaction with daily life, thereby further boosting their subjective well-being. At the same time, the improvement in self-esteem resulting from outdoor physical activity may be closely related to increased self-efficacy. During physical activity, college students can gradually build self-efficacy by achieving exercise goals, overcoming physical challenges, and perceiving positive changes in their physical condition. Self-efficacy is considered one of the key psychological mechanisms through which physical activity influences mental health (Li and Huang, 2025). Therefore, outdoor physical activity not only improves emotional well-being through the exercise itself but may also further promote the enhancement of college students’ self-esteem by boosting self-efficacy and body self-perception (Yu et al., 2024).

Outdoor physical activity may effectively alleviate academic burnout among college students. Academic burnout typically manifests as emotional exhaustion, learning-related apathy, and a diminished sense of accomplishment, and its development is often closely associated with sustained academic pressure, depletion of psychological resources, and insufficient recovery (Schaufeli et al., 2002; Zhang et al., 2025). On the one hand, outdoor physical activity promotes physical and mental relaxation, thereby alleviating emotional exhaustion caused by prolonged academic stress. On the other hand, it disrupts sedentary routines and monotonous study environments, allowing college students to temporarily disengage from high-intensity cognitive demands and facilitating the restoration of psychological resources (Xu, 2025; Puhakka, 2021). Furthermore, the sense of accomplishment and positive feedback derived from physical activity may enhance self-efficacy, counteract feelings of helplessness and frustration caused by academic stress, and thereby further reduce academic burnout (Chen et al., 2022; Pan et al., 2024). Future research should employ longitudinal designs aligned with the academic calendar to compare intervention effectiveness across key stages of college life, including the transition into university, regular teaching periods, examination weeks, and the graduation-to-employment transition. Such studies should also examine whether academic workload moderates the effects of outdoor physical activity on academic burnout.

There is currently no consensus regarding the effects of outdoor exercise on anxiety and attention. This lack of consensus may be attributable to differences in exercise type, exercise intensity, intervention duration, characteristics of the outdoor environment, and participants’ baseline conditions. On the one hand, activities such as water sports, running, and training in green spaces may provide both substantial physical stimulation and sufficient exposure to natural environments, making them more likely to exert positive effects through physiological activation, emotional regulation, and attentional restoration. On the other hand, low-intensity or single-session interventions, such as outdoor walking or strength training, may involve relatively insufficient intervention doses, or the restorative qualities of the outdoor environments may be insufficiently pronounced, making it difficult to consistently improve anxiety or attention. Furthermore, differences across studies in measurement tools for anxiety and attention, sample characteristics, and intervention settings may also contribute to inconsistent findings. Future studies should use standardized anxiety measures and objective cognitive tasks and report participants’ baseline anxiety, mental health status, physical activity levels, and prior exposure to natural environments to reduce the influence of sample heterogeneity. At the same time, the specific roles of different forms of outdoor physical activity should be further clarified, and the potential mechanisms by which outdoor physical activity improves anxiety and attention in college students should be explored in depth, taking into account exercise dose, degree of exposure to natural environments, and psychocognitive indicators.

Outdoor physical activity may positively influence both physical activity levels and physical health among college students. It can enhance students’ motivation to participate in exercise, increase the frequency and duration of physical activity, and thereby promote an overall increase in physical activity levels (Baceviciene and Jankauskiene, 2021). Previous studies have shown that physical activity in natural environments helps improve mental health, enhance well-being, and provide college students with opportunities for emotional restoration (Coventry et al., 2021). Improved mental health may further reduce physical and psychological stress, improve sleep quality and quality of life, and strengthen individuals’ motivation to engage in healthy behaviors, thereby indirectly promoting physical health. In addition, outdoor physical activity can increase physical activity levels, and adequate physical activity contributes to improved cardiopulmonary function, musculoskeletal health, and energy metabolism while reducing the risk of chronic diseases (Han and Wang, 2018; Garber et al., 2011; Bull et al., 2020). Therefore, outdoor physical activity may promote physical health among college students by improving mental health and increasing physical activity levels.

## 5 Strengths and limitations of the study

This study is the first to systematically examine the effects of outdoor physical activity on the physical and mental health of college students, and its findings may serve as an important reference for mental health interventions and the design of physical activity programs for this population. This study has several strengths. First, literature searches and data extraction were conducted in strict accordance with the PRISMA guidelines, and quality assessment tools were used to evaluate the methodological quality of the included studies, thereby enhancing the standardization of the research process and the reliability of the findings. Second, by focusing on college students as a specific population, this study provides findings with strong relevance and practical value. Finally, this study examined not only mental health indicators, such as depression, anxiety, stress, mood, well-being, and academic burnout, but also physical activity and physical health-related outcomes. Consequently, it provides a comprehensive understanding of the potential pathways through which outdoor physical activity may promote health among college students by simultaneously improving mental health and increasing physical activity levels. Despite its detailed review and discussion, this study has several limitations. First, the included studies varied substantially in intervention design, exercise intensity, intervention duration, type of outdoor environment, and measurement tools, resulting in considerable heterogeneity. This limits direct comparisons across studies and affects the precise estimation of overall effects. Second, the number of studies addressing certain outcomes remains limited, particularly those related to physical activity levels and physical health, for which the available evidence is relatively scarce. Therefore, the interpretation of how outdoor physical activity promotes physical health among college students should be made with caution. Third, some included studies had small sample sizes, short intervention periods, and no long-term follow-up, making it difficult to assess the sustained effects of outdoor physical activity on the physical and mental health of college students. Overall, this study provides a relatively systematic evidence base for promoting physical and mental health among college students through outdoor physical activity. However, existing evidence remains insufficient regarding physical health outcomes, long-term intervention effects, and underlying mechanisms. Future research should adopt more rigorous randomized controlled designs, clearly report exercise intensity, frequency, duration, and outdoor environmental characteristics, and combine subjective and objective measures with long-term follow-up to further elucidate the mechanisms by which outdoor physical activity influences mental health, physical activity levels, and physical health among college students. Finally, although the search strategy was informed by previous relevant research and included terms related to physical activity, physical and mental health, it provided more comprehensive coverage of mental health outcomes than of physical health outcomes. Specifically, the search may not have captured the full range of terms relating to physical function, cardiorespiratory health, body composition, sleep, fatigue, physiological indicators, and health behaviors. Consequently, some studies on physical health outcomes may not have been identified, and there may be a certain degree of omission in the evidence regarding physical health. Future research should develop a more systematic and detailed thesaurus of search terms for physical health outcomes and combine subject headings, free-text search and citation tracking to enhance the comprehensiveness of the search.

## 6 Conclusion

The results of this study indicate that outdoor physical activity may improve both physical and mental health among college students. Overall, outdoor physical activity showed positive effects on mental health, particularly in alleviating depressive symptoms, reducing stress levels, enhancing mood, vitality, well-being, and self-esteem, and reducing academic burnout. In addition, outdoor physical activity may increase physical activity levels and promote physical health among college students. However, this study also found that the effects of outdoor physical activity on anxiety and attention were inconsistent. Overall, outdoor physical activity represents an important approach to promoting the physical and mental health of college students and may provide guidance for the development of physical education curricula, campus health promotion programs, and mental health interventions. Future research should adopt more rigorous randomized controlled designs and incorporate objective measures of physical activity and physical health to further clarify the mechanisms and long-term effects of different forms of outdoor physical activity, exercise dose, and environmental characteristics on the physical and mental health of college students.

## Data Availability

The data used and /or analyzed in the current study are available from the first author on reasonable request.
